# BRCA1 foci test as a predictive biomarker of olaparib response in ovarian cancer patient-derived xenograft models

**DOI:** 10.3389/fphar.2024.1390116

**Published:** 2024-06-25

**Authors:** Federica Guffanti, Ilaria Mengoli, Maria Francesca Alvisi, Giulia Dellavedova, Raffaella Giavazzi, Robert Fruscio, Eliana Rulli, Giovanna Damia

**Affiliations:** ^1^ Laboratory of Preclinical Gynaecological Oncology, Experimental Oncology Department, Istituto di Ricerche Farmacologiche Mario Negri IRCCS, Milan, Italy; ^2^ Laboratory of Methodology for Clinical Research, Clinical Oncology Department, Istituto di Ricerche Farmacologiche Mario Negri IRCCS, Milan, Italy; ^3^ Laboratory of Cancer Metastasis Therapeutics, Experimental Oncology Department, Istituto di Ricerche Farmacologiche Mario Negri IRCCS, Milan, Italy; ^4^ Clinic of Obstetrics and Gynecology, Department of Medicine and Surgery, San Gerardo Hospital, University of Milan Bicocca, Monza, Italy

**Keywords:** ovarian cancer, BRCA1 foci, homologous recombination deficiency testing, biomarker, drug resistance

## Abstract

Standard therapy for high-grade ovarian carcinoma includes surgery followed by platinum-based chemotherapy and poly-ADP ribose polymerase inhibitors (PARPis). Deficiency in homologous recombination repair (HRD) characterizes almost half of high-grade ovarian carcinomas and is due to genetic and epigenetic alterations in genes involved in HR repair, mainly *BRCA1/BRCA2,* and predicts response to PARPi. The academic and commercial tests set up to define the HRD status of the tumor rely on DNA sequencing analysis, while functional tests such as the RAD51 foci assay are currently under study, but have not been validated yet and are available for patients. In a well-characterized ovarian carcinoma patient-derived xenograft platform whose response to cisplatin and olaparib, a PARPi, is known, we assessed the association between the BRCA1 foci score, determined in formalin-fixed paraffin-embedded tumor slices with an immunofluorescence technique, and other HRD biomarkers and explored the potential of the BRCA1 foci test to predict tumors’ response to cisplatin and olaparib. The BRCA1 foci score was associated with both tumors’ HRD status and RAD51 foci score. A low BRCA1 foci score predicted response to olaparib and cisplatin, while a high score was associated with resistance to therapy. As we recently published that a low RAD51 foci score predicted olaparib sensitivity in our xenobank, we combined the two scores and showed that the predictive value was better than with the single tests. This study reports for the first time the capacity of the BRCA1 foci test to identify HRD ovarian carcinomas and possibly predict response to olaparib.

## Introduction

High-grade ovarian carcinoma (HGOC) represents the most diffused and lethal gynecological malignancy in the Western countries (Sung et al., 2021). Standard therapy includes surgery followed by platinum-based chemotherapy and poly-ADP ribose polymerase inhibitors (PARPi), both extremely active in homologous recombination-deficient (HRD) tumors (Lord and Ashworth, 2016). HRD status characterizes almost half of HGOC and is due to genetic and epigenetic alterations in genes involved in HR repair, mainly *BRCA1/BRCA2* (Konstantinopoulos et al., 2015). Tumor HRD is among the most highly regarded predictive biomarkers of PARPi response and is currently determined with commercial and academic molecular assays (Mangogna et al., 2023). The RAD51 foci test is emerging as a predictive tool for platinum agents and PARPi sensitivity (Pellegrino et al., 2022), but all these assays may not be sufficient to capture the complexity of the DNA repair-related mechanisms underlying HRD.

In this study, we assessed the predictive potential of the BRCA1 foci test, similar to the RAD51 foci assay, in a large collection of 55 well-characterized ovarian carcinoma patient-derived xenograft (OC-PDX) models (Supplementary Table S1), whose response to cisplatin (DDP) and olaparib, a PARPi, is known (Ricci et al., 2014).

## Materials and methods

### Ovarian carcinoma xenobank

Our collection of OC-PDX (xenobank) has been established as already detailed in Ricci et al. (2014) and Guffanti et al. (2020). This xenobank consists of subcutaneously (s.c.) and intraperitoneally (i.p.) transplanted models. Some of them derive from relapsing platinum-treated tumors; however, none of them comes from tumors pre-treated with PARPi. The xenobank also includes five DDP-resistant models, obtained through multiple *in vivo* DDP treatment cycles (Ricci et al., 2019). Forty-seven PDXs have been characterized for DDP and thirty for olaparib response, as previously described in Ricci et al. (2014) and Guffanti et al. (2022).

### BRCA1 foci test

To quantify BRCA1 nuclear foci, we used an immunofluorescence (IF)-based method similar to that described in (10). In brief, 3-µm-thick formalin-fixed paraffin-embedded (FFPE) tumor sections were deparaffinized and antigens were retrieved using DAKO antigen retrieval buffer pH 9.0 (Agilent DAKO). The primary and secondary antibodies used to detect BRCA1 and geminin were mouse anti-BRCA1 sc-6954 (Santa Cruz Biotechnology) (diluted at a ratio of 1:50); geminin polyclonal antibody 10802-1-AP (Proteintech Group) (1:400); goat anti-mouse IgG (H + L) Cross-Adsorbed Secondary Antibody, Alexa Fluor™ 568 (Thermo Fisher Scientific) (1:500) for BRCA1 foci; and goat anti-rabbit IgG (H + L) Cross-Adsorbed Secondary Antibody, Alexa Fluor™ 488 (Thermo Fisher Scientific) (1:500) for geminin. Slides were mounted using ProLong™ Gold Antifade Mountant with DNA Stain DAPI (Invitrogen). Microphotographs of IF-stained samples were acquired using a Nikon A1 confocal microscope, with the 60 ×/1.27 WI Plan APO IR, ∞ 0.15/0.19 WD 0.18-0.16 objective (Nikon) and analyzed using ImageJ FIJI software (Schindelin et al., 2012) by applying an in-house macro tool that allows us to discriminate the three different channels (i.e., blue for DAPI, green for geminin positive nuclei, and red for the nuclear foci) within the same image. The percentage of BRCA1 nuclear foci-positive cells was quantified blind by manually selecting the geminin-positive tumor cells and quantifying how many expressed at least five foci per nucleus (named“% BRCA1+/GMN+ cells” or “BRCA1 foci score”). At least 100 geminin-positive tumor cells in 10 different areas of the tissue section were analyzed. The RAD51 foci scores in the same PDX FFPE tumor samples have already been published (Guffanti et al., 2022).

### Statistical analyses

The Kruskal–Wallis test was used to analyze the association between % BRCA1+/GMN+ cells, considered a continuous variable, and HR biomarkers and responses to DDP and olaparib, treated as categorical variables. The receiver operating characteristic (ROC) curve and the Youden index were used to define the BRCA1 foci cut-off to better discriminate the PDXs on their response to therapy (sensitive vs*.* resistant), considering equal weight to sensitivity and specificity. The ROC curve and area under the ROC curve (AUC) analysis were also carried out for the model combining BRCA1 and RAD51 foci cut-off for olaparib response. The positive predictive values (PPVs) and negative predictive values (NPVs) were calculated considering response to therapies as dichotomous variables: responsive as positive and resistant as negative. Statistical analysis and graphs were done using GraphPad Prism 9.5.1 (GraphPad Software).

## Results

**FIGURE 1:**
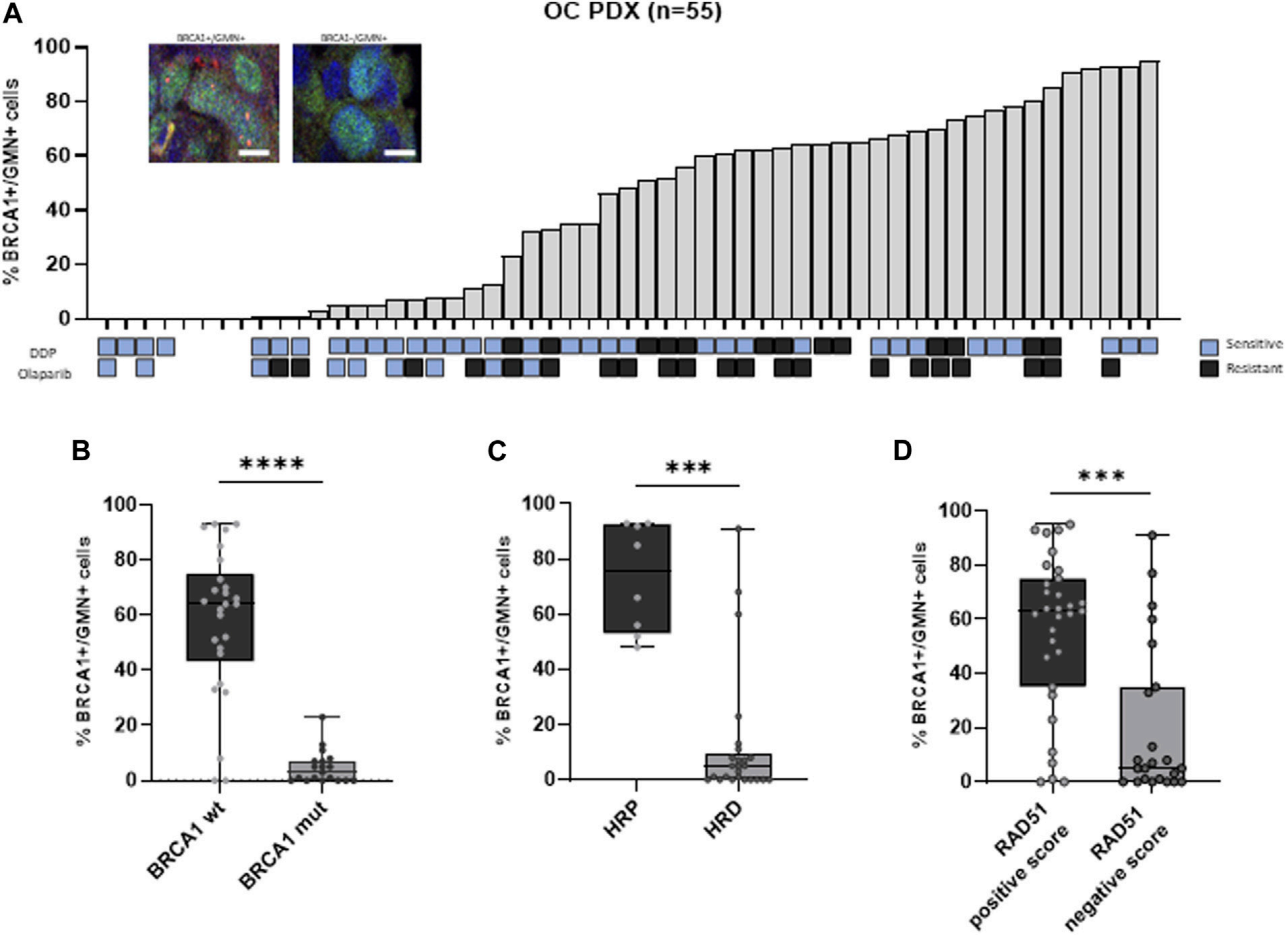
**(A)** Immunofluorescence of geminin-positive (GMN+) nuclei (in green) (at least 100 per samples were evaluated) expressing or not BRCA1 foci (red nuclear dots). The histogram shows the percentage of BRCA1 foci-positive cells (% BRCA1+/GMN + cells) in the 55 OC-PDX models analyzed. When available, the sensitivity to DDP and olaparib is reported below the graph (blue squares = resistant; gray squares = sensitive). **(B)** Association between BRCA1 foci-positive cells and BRCA1 mutational status of the PDXs (Kruskal–Wallis test, ****: *p* < 0.0001). **(C)** Association between BRCA1 foci-positive cells and HR status (Kruskal–Wallis test, ***: *p* < 0.001). **(D)** Association between BRCA1 foci-positive cells and RAD51 foci-positive cells previously quantified in the same PDXs (Kruskal–Wallis test, ***: *p* < 0.001).

**FIGURE 2:**
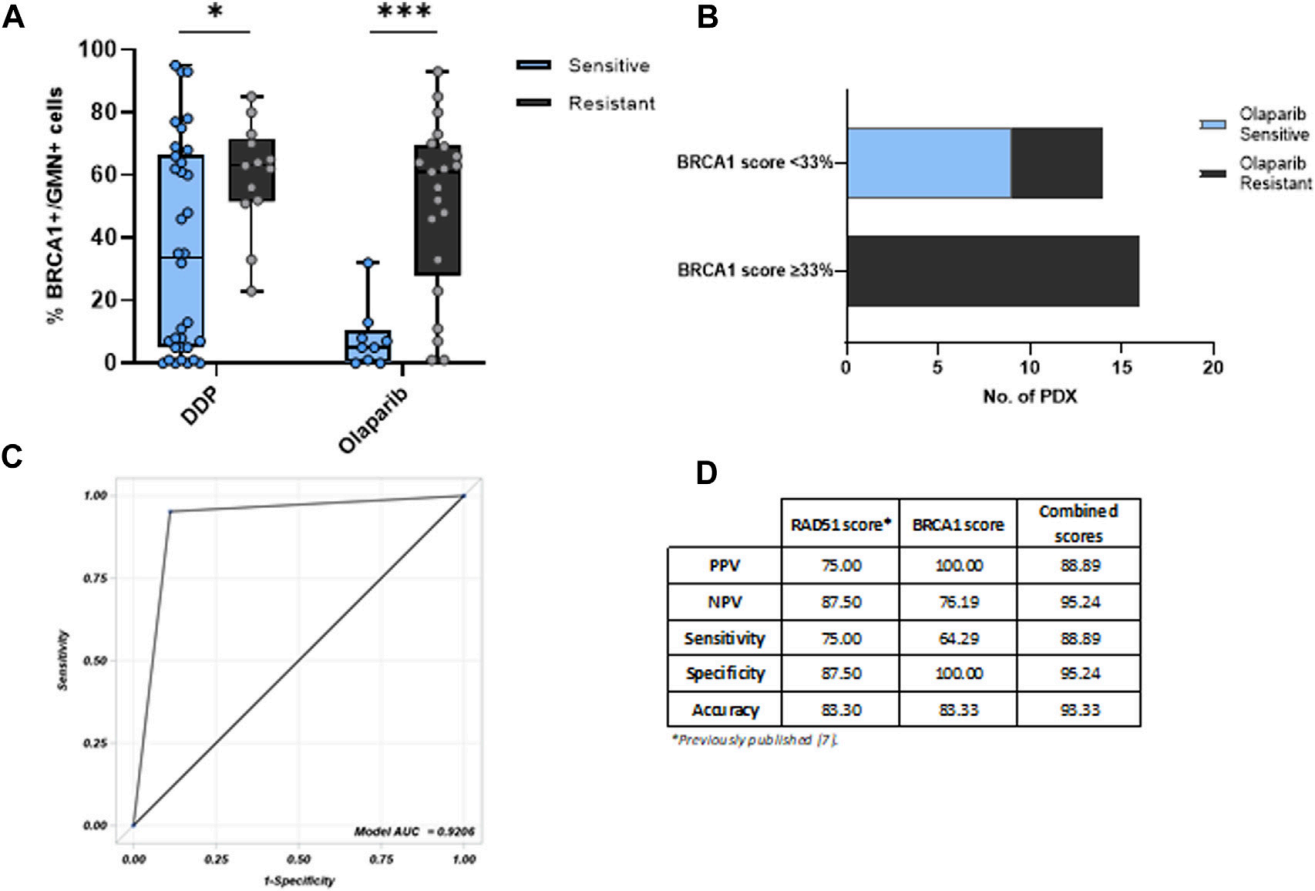
**(A)** BRCA1 foci-positive score associated with the OC-PDX response to cisplatin (DDP) and olaparib. The % BRCA1+/GMN + cells is significantly lower in PDXs sensitive to both DDP and olaparib, compared with those resistant to therapy (Kruskal–Wallis test, *: *p* < 0.05; ***: *p* < 0.001). **(B)** BRCA1 foci cut-off of 33% and OC-PDX stratification for olaparib response. **(C)** ROC curve of BRCA1/RAD51 foci-combined scores and olaparib response in OC-PDXs. **(D)** Summary table with positive predictive values (PPVs) and negative predictive values (NPVs) of the two single tests and of the combined model.

The basal RAD51 foci score in FFPE tumors from our xenobank was previously published (Castroviejo-Bermejo et al., 2018), and in the same 55 OC-PDXs, the percentage of BRCA1+/GMN+ cells was assessed. It ranged from 0% to 95% with a median of 48% and a mean value of 41% ± 0.33 (st. dev.) (Figures 1A; Supplementary Table S1). BRCA1 foci scores were significantly lower in 1) *BRCA1*-mutated PDXs (*n* = 19) than in wild-type xenografts (n = 26) (median of BRCA1+/GMN+ cells = 2.5% (min–max 0%–23%) vs*.* 64.1% (0%–93%) (Kruskal–Wallis test, *p* < 0.0001, Figures 1B); 2) HRD PDXs, classified on the basis of the presence of *BRCA1/BRCA2* pathogenic mutations, on the HRDetect score and *BRCA1* promoter hypermethylation (Guffanti et al., 2022) (n = 25) than in HR-proficient models (*n* = 8) (median = 5% (0%–91%) vs*.* 75.3% (48%–93%)) (*p* = 0.0002, Figures 1C); and 3) RAD51 foci-negative PDXs (RAD51 foci score ≤10%, *n* = 23) (Castroviejo-Bermejo et al., 2018; Cruz et al., 2018) than in the RAD51 foci-positive PDXs (RAD51 foci score >10%, *n* = 31) (median = 5% (0%–91%) vs*.* 63% (0%–95%)) (*p* = 0.0002, Figures 1D). All these data strongly suggest the BRCA1 foci score as a biomarker of HRD.

We then analyzed the relationship between the BRCA1 foci score and response to therapy since almost all OC-PDXs are characterized and classified as sensitive or resistant based on their *in vivo* response to DDP and olaparib drugs (Figures 1A; Supplementary Table S1) (Ricci et al., 2014; Guffanti et al., 2020). Tumors responsive to olaparib (*n* = 9) and DDP (*n* = 34) expressed significantly lower levels of % BRCA1+/GMN+ cells than the resistant PDXs (olaparib-resistant *n* = 21 and DDP-resistant *n* = 13) (Figures 2A). DD-sensitive and DDP-resistant PDXs had medians of 33.5% (0%–95%) and 63% (23%–85%) of BRCA1 foci-positive cells (*p* = 0.033), respectively, while the olaparib-sensitive models had a median of 5% (0%–32%) and the olaparib-resistant models, 61% (1%–93%) (*p* = 0.0006) (Figures 2A).

A cut-off of 33% of the BRCA1 foci score clustering sensitive and resistant tumors to olaparib (i.e., < 33% associated with sensitivity and ≥33% with resistance) was derived from the Youden index in order to optimize the discriminative capacity of the receiver operating characteristic (ROC) curve, whose area under the curve (AUC) was 0.8995 (Supplementary Figure S1A). Unfortunately, for DDP response, the ROC curve analysis did not allow the identification of a discriminative cut-off (Supplementary Figure S1B), probably due to the fact that the repair of DDP-induced DNA damage not only relies on HR but also on other repair mechanisms such as nucleotide excision repair (NER) and the Fanconi anemia pathway (FA) (Mesquita et al., 2019; Li et al., 2022; Zhang et al., 2022).

Applying the 33% cut-off, all the olaparib-sensitive PDXs clustered (all had a negative score <33%), and all the olaparib-resistant PDXs had positive BRCA1 foci scores (≥33%) (Fisher test, *p* = 0.0001), except 5 out of 21 olaparib-resistant PDXs, which had a negative score and all of them were *BRCA1*-mutated HGOC (Figures 2B). These data suggest that resistance might be due to factors outside *BRCA1*, such as the loss of 53BP1, RIF1 and REV7 (parts of the Shieldin complex), or DYNLL1 (Xu et al., 2015; Nacson et al., 2018; Swift et al., 2023).

When we combined the two RAD51 and BRCA1 foci scores with their reported cut-off values (10% for RAD51 foci (Castroviejo-Bermejo et al., 2018) and 33% for BRCA1 foci test), the ROC curve deriving from the combination of these two assays had higher AUC = 0.9206 (Figures 2C) than the single BRCA1 and RAD51 foci assays. Considering the predictive values of the combined test, it showed a good positive predictive value compared to the single tests (PPV = 88.89% vs*.* 75% and 100% of RAD51 foci and BRCA1 foci tests, respectively), and a superior negative predictive value (NPV = 95.24% for the combined scores vs*.* 87,50% and 76,19% for RAD51 and BRCA1 tests, respectively) with an improved accuracy (93,33% of the combined assay vs*.* 83.30% and 83.33% of the single assay) (Figures 2D).

## Discussion

**FIGURE 3:**
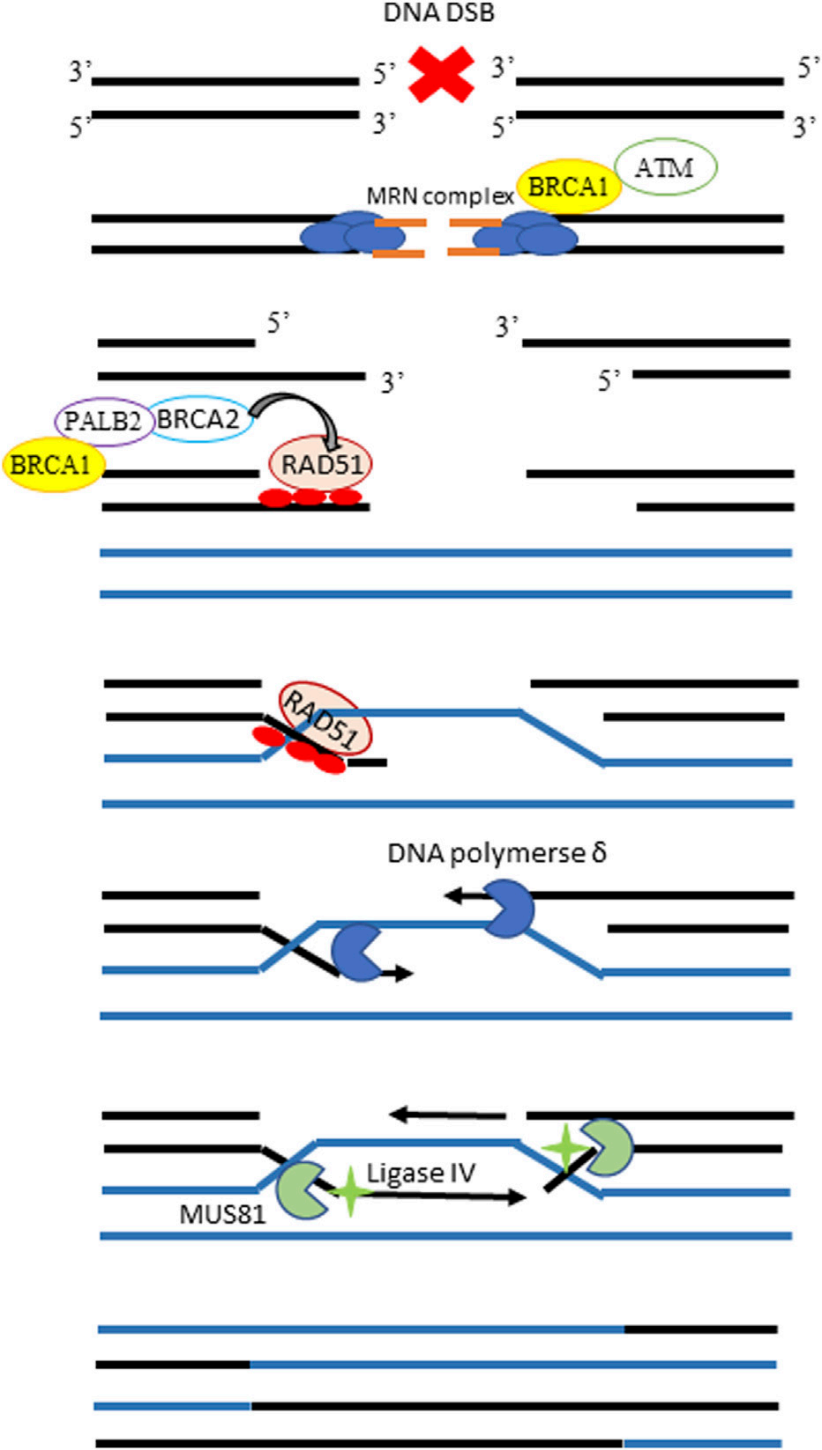
Schematic workflow of the homologous recombination pathway. In the presence of a DNA DSB, the MRN complex detects and binds the broken DNA ends, recruiting BRCA1 and the ataxia-telangiectasia kinase (ATM) proteins. ATM phosphorylates and activates BRCA1 on the DSB site, promoting the end resection and leading to the exposure of two ssDNA regions, which overhang on either side of the DSB. The BRCA2 protein is then recruited at the damaged site by BRCA1, and their interaction is mediated by PALB2. The central player of HR repair, the single strand-binding protein RAD51 which forms visible nuclear foci, is loaded onto the 3’single-strand overhangs by BRCA2 and guides strand invasion to have the homologous sequences in the intact sister chromatid as a substrate for repair. DNA polymerase uses the homologous sister chromatid as a template and uses the ssDNA as a primer to synthesize new DNA sequences. The final steps involve endonuclease MUS81 and ligase IV to solve this complex structure and complete the solution of DSB.

DNA double-stranded breaks (DSBs) are the most lethal cytotoxic lesions, and several DNA repair pathways with different degrees of fidelity exist to cope with this kind of lesions (for an update review see (Scully et al., 2019)). HR repair represents the most accurate pathway, and its schematic workflow is depicted in Figure 3. Functional inactivation of HR, due to mutations or hypermethylation of genes involved in the pathway, a condition known as *BRCAness* or HR deficiency (HRD), characterizes half of HGOC (Konstantinopoulos et al., 2015) and accounts for the high genomic instability in this tumor type. At the same time, the lack of a functional HR also renders HGOC, particularly sensitive to platinum- and PARPi-based therapies (Lord and Ashworth, 2016; Pilié et al., 2019). The assessment of tumor HR status could help not only in a better stratification of patients undergoing first-line therapy but also in the identification of cases becoming drug resistant. Genomic tests have been developed to identify HRD tumors (Ngoi and Tan, 2021). These strategies generally rely on massive sequencing technologies (i.e., next-generation sequencing or whole-genome sequencing) aimed at identifying mutations in HR genes, as well as genomic signatures driven by defects in HR repair. However, the major limitation to these tests is that they just provide peculiar genomic scars related to a prior HRD exposure condition, but they do not give information on the current HR functionality (Fuh et al., 2020). HR functional assays conceived to overcome these limitations are urgently requested and are under development. We have already reported in our well-characterized xenobank the role of RAD51 foci score as a surrogate marker of the functional HR and predictive biomarker of olaparib response. The basal level of the RAD51 foci score in untreated FFPE tumor samples has been previously advocated as a marker of functional HR and shown to predict olaparib response in breast cancer PDXs (Castroviejo-Bermejo et al., 2018; Cruz et al., 2018) and, more recently, to predict response to platinum-based neoadjuvant therapy in a retrospective study (Llop-Guevara et al., 2021). With a similar IF-based assay, in this manuscript, we investigated the basal BRCA1 foci score as a potential functional biomarker of the HRD status and predictive marker of response to therapy in our OC xenobank.

We used this method to evaluate the presence in FFPE samples of BRCA1 foci in proliferating (geminin positive) tumor cells because HR is restricted in cells in the S-phase, without the need to induce external DNA damage. Notably, this latter condition unequivocally favors the application of the method in a clinical setting where FFPE tumor biopsies are routinely collected. The IF-BRCA1 foci method we used here relies on the use of a confocal microscope, giving the possibility to acquire different images for each sample and allowing the scoring in a semi-automatic way by combining the use of a software application for imaging analyses with the manual quantification of foci-positive cancer cells. Our method allows keeping images saved for further/subsequent analyses and reduces inter-operator assessment variability.

In this way, 55 OC-PDXs from our xenobank (Ricci et al., 2014; Guffanti et al., 2022) were evaluated for the basal expression of cells positive for BRCA1 foci and a heterogeneous pattern of expression was found (from none to high levels). To evaluate the capability of the BRCA1 foci test to correlate with the HRD status of tumors, we first compared BRCA1 foci levels of the PDXs with their *BRCA* mutational status, *BRCA1* promoter methylation levels, the HRDetect score (a genomic HRD assay (Davies et al., 2017)), and with the RAD51 foci levels, being all well-established HRD biomarkers. We found a statistically significant association with the mutational status of the *BRCA1* gene; in particular, those PDXs carrying *BRCA1* pathogenic mutations had a lower number of BRCA1 foci score than the wild-type PDXs. Similarly, those tumors defined as HRD (based on the presence of *BRCA1/2* mutations, *BRCA1* promoter hypermethylation, and HRDetect score ≥0.7 (Davies et al., 2017)) showed lower BRCA1 foci score than HR-proficient tumors (*BRCA1/2* wild-type along with no-hypermethylated *BRCA1* promoter and HRDetect score <0.7). We found only three PDXs classified as HRD expressing high levels of BRCA1 foci: two of them are *BRCA2*-mutated (i.e., MNHOC241 and MNHOC280, Supplementary Table S1), so they might correctly form BRCA1 foci, while the third model is a *BRCA1/2* wild-type tumor (i.e., MNHOC135) but classified as HRD based on the HRDetect genomic test. However, in all the cases, the HR repair cannot proceed further, as also suggested by the low RAD51 foci levels detected in two of these models, so they can be defined as HRD tumors despite the presence of BRCA1 foci.

We found a statistically significant association between RAD51 and BRCA1 foci scores, with most of the tumors having less than 10% of the RAD51 score also having a low amount of BRCA1 foci score, but some exceptions exist. Unfortunately, the HRD status of some of these tumors has not yet been defined. These latter discrepancies could be explained by the fact that RAD51 acts downstream of BRCA1 in the HR pathway (Figure 3 and reviewed in Prakash et al. (2015)), and there could be different factors and/or alterations impairing HR by affecting just one of the two proteins.

As a whole, these preliminary results support the use of the BRCA1 score to identify HRD tumors. We previously reported in our OC preclinical models that low expression levels of RAD51 foci-positive cells predicted a response to olaparib but not to DDP (Guffanti et al., 2022). Even if we found a statistically significant association between the BRCA1 score and both DDP and olaparib responses (i.e., lower foci score predicting high drug response), the association was less robust with DDP response. This could be due to the fact that HR deficiency is not the only determinant of DDP response; in fact, NER and/or FA pathways are involved in the removal of the platinum-DNA adducts but not in the repair of PARPi-induced damages (Mesquita et al., 2019; Li et al., 2022; Zhang et al., 2022). In this regard, a detailed analysis of the role of NER and FA pathways is under study in our models. In this study, we tried to establish a cut-off able to discriminate DDP-sensitive and DDP-resistant PDXs; however, the analysis of the ROC curve revealed that the best cut-off (i.e. 24%) was not sufficiently robust to discriminate such response, suggesting that the basal BRCA1 foci score alone may not be an ideal biomarker to predict the DDP response in ovarian carcinomas.

Regarding olaparib response, the analysis of the ROC curve provided a cut-off of 33% of the BRCA1 foci score, which was able to distinguish tumors as responsive and non-responsive to olaparib. Most of the PDXs with a percentage of BRCA1 foci-positive cells lower than 33% were responsive, while all the PDXs with a BRCA1 foci score higher than 33% were resistant to olaparib.

However, by setting the cut-off of 33%, we observed some discrepancies. Specifically, 5 out of 21 tumors (24%) could be misidentified. For this reason and also considering that both BRCA1 and RAD51 are involved at different levels of the HR pathway, we evaluated if their combined analysis could improve the ability to predict olaparib response. By combining the cut-off of both single tests (i.e. 10% and 33%), the negative predictive value, the accuracy, and sensitivity improved while maintaining a good positive predictive value. So, according to this combined model, it is sufficient to have at least one of the two markers at higher levels than the relative threshold in order to be classified as the tumor resistant to olaparib; on the contrary, when both RAD51 and BRCA1 foci are expressed at lower levels than their relative cut-offs, the tumors are likely to be sensitive to the treatment.

For the first time, this study reports the potential role of the BRCA1 foci score to identify HRD ovarian carcinomas. Although low BRCA1 scores were enriched in PDX models responsive to both DDP and olaparib, a cut-off value was not defined to cluster-sensitive and cluster-resistant DDP tumors. On the contrary, a cut-off value of 33% was found to discriminate-sensitive (<33%) from resistant PDXs (≥33%). The olaparib predictivity of this score was improved when it was combined with the RAD51 foci score.

This study has some limitations. Indeed, even if our ovarian PDXs well mimic human OC, they do not fully capture its complexity. In particular, nude mice lack part of the immune system that may affect the response to therapy in OC patients (Ovarian Tumor Tissue Analysis Consortium et al., 2017; Park et al., 2023). Nevertheless, we have enhanced the performance of the BRCA1 foci assay by setting up a semi-automatic method for the analysis of nuclear foci, greatly reducing possible inter-operator-related bias. However, a total automated protocol, preferably based on immunohistochemistry rather than immunofluorescence staining, would be required for both BRCA1 and RAD51 foci evaluation to be fully translated into the clinical pathological routine.

Further studies are ongoing to corroborate these results in other well-defined patient cohorts, whose HRD status and response to PARPi-based therapy are available. Notably, considering that other tumor types such as prostate cancer, pancreatic carcinoma, breast cancer, and mesothelioma may harbor DNA defects (Golan et al., 2019; Abida et al., 2020; Chopra et al., 2020), the application of BRCA1/RAD51 foci assays could be enlarged to a wider population of oncological patients, providing cost-effective predictive tools to better tailor patients’ therapy.

## Data Availability

The raw data supporting the conclusion of this article will be made available by the authors, without undue reservation.

## Supplementary material

SUPPLEMENTARY FIGURE S1
**(A)** ROC curve of the BRCA1 foci score and olaparib response. **(B)** ROC curve of the BRCA1 foci score and DDP response.

SUPPLEMENTARY TABLE S1 OC-PDXs used in this study with their pharmacological and molecular characteristics.
