# Supplementary figures and images for: BRCA1 foci test as a predictive biomarker of olaparib response in ovarian cancer patient-derived xenograft models
Source: Front Pharmacol. 2024 Jun 25;15:1390116. doi: 10.3389/fphar.2024.1390116 (PMC11234799; doi:10.3389/fphar.2024.1390116)

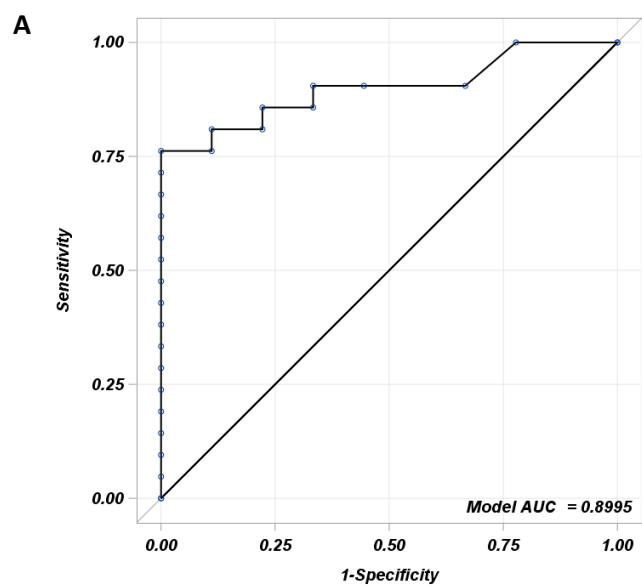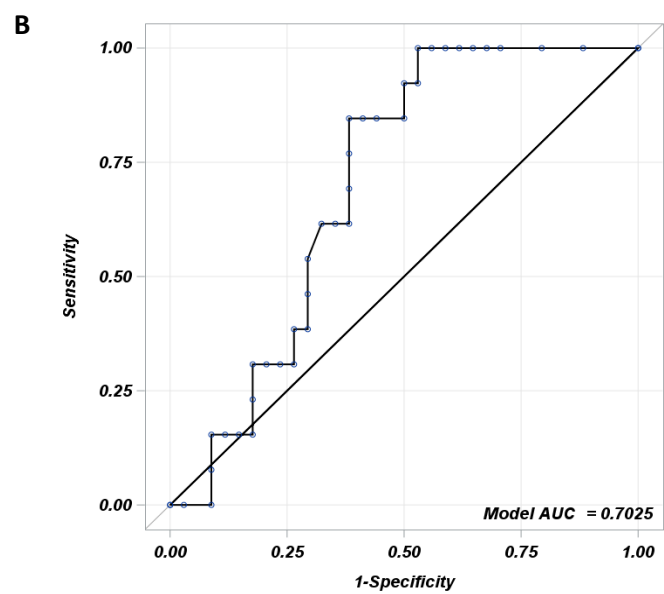

**Supplementary Fig. 1**

Supplement: Supplementary file 1 [file DataSheet1.PDF]
